# Effects of counseling professional ethics principles on midwifery professional codes of ethics compliance and applicability rate among midwives in community health centers: a randomized clinical trial in Iran

**DOI:** 10.11604/pamj.2020.35.139.20702

**Published:** 2020-04-29

**Authors:** Soheila Shahabnia, Razieh Lotfi, Mitra Rahimzadeh, Mansoureh Yazdkhasti, Zahra Mehdizadeh Tourzani

**Affiliations:** 1 Student in Midwifery Counseling, Student Research Committee, Alborz University of Medical Sciences, Karaj, Iran; 2 Midwifery and Reproductive Health Department, School of Medicine, Alborz University of Medical Sciences, Karaj, Iran; 3 Social Determinants of Health Research Center, Alborz University of Medical Sciences, Karaj, Iran

**Keywords:** Ethics, professional, codes of ethics, reproductive health, counseling

## Abstract

**Introduction:**

Compliance with ethical principles is regarded as one of the key components in providing services in midwifery profession. This study was to evaluate the effects of counseling professional ethics principles on midwifery professional codes of ethics compliance and applicability rate among midwives working in community health centers in the city of Karaj, Iran.

**Methods:**

This randomized controlled trial (RCT) was conducted in 2018 on a total number of 84 eligible midwives in two intervention and control groups, selected through multistage sampling method. The intervention group took part in six counseling sessions but the control group only received a training manual. Both groups then completed the Self-Reporting Questionnaire of Ethical Codes of Reproductive Health Providers (including 95 items in 14 domains) at three time points (before, immediately, and four weeks after intervention). Finally, the data were analyzed using the IBM SPSS Statistics (version 22) software via descriptive and inferential statistics.

**Results:**

The findings showed that level of compliance and applicability rate in all 14 domains of midwifery professional codes of ethics were higher in the intervention group (after intervention) than those in the control group and trend of time changes in mean level of compliance and applicability rate of codes of ethics during the three time points were significantly different between both groups (p < 0.001).

**Conclusion:**

Given the effectiveness of counseling professional ethics principles on midwifery professional codes of ethics compliance and applicability rate among the midwives working in community health centers, designing and applying this counseling approach was recommended to improve quality of reproductive health care services.

## Introduction

Ethics, with a long history of over 2500 years all over the world [1], refers to principles and values governing individuals and collective behaviors, and determining if something is good or bad [2]. Ethics has always been a subject of debate among scientists, philosophers, and religious scholars, so it has become more problematic to distinguish ethical considerations from unethical ones [3]. The approach of today´s world can be also seen as a return to rationality and human ethics [4]. Therefore, ethics can be considered as the center of future world developments [5]. Medical ethics is also a branch of professional ethics, accounting for ethical and specialized codes practiced by medical professionals [6]. In this respect, the concept of codes of medical ethics encompasses ethical principles including beneficence, non-maleficence, respect for autonomy, and justice. Furthermore, ethical rules contain privacy, honesty, loyalty, and respect for privacy [7]. Among various medical professions, midwifery has particular holiness and sensitivity, so it is imperative to pay attention to its ethical foundations [6]. Midwives also play important roles in counseling and educating women, families, and communities in terms of health. Furthermore, they are responsible for maintaining and promoting maternal and child health [8].

The International Confederation of Midwives (ICM) was established in 1996 with a meeting of midwives from 72 countries to achieve the goal of promoting health status of women and children and improving quality of midwifery care services. The members of the ICM have thus far worked to fulfill the stated goals in formulating midwifery codes of ethics [9]. Accordingly, the Iranian Ministry of Health and Medical Education (MHME) developed and published 85 midwifery professional codes of ethics in 6 domains based on working conditions and dominant culture in 2013 with the aim of promoting quality of midwifery care services in this country [10]. It should be noted that midwives need to be well aware of ethical and legal scopes of their duties because of the wide variety of services offered in the broad field of reproductive health [11]. A number of studies have revealed that some aspects of professional ethics have been poorly observed. In this respect, Rahimparvar *et al*. (2014) found that level of compliance with professional codes of ethics in midwives in the city of Tehran was generally moderate [9]. Mosalanejad and Ghorbanifar (2013) also reported that health care staff had no good levels of compliance with ethical principles, and they had even performed poorly on commitment and secrecy [12]. Moreover, Turkmen *et al*. (2015) suggested that most nurses working in pediatric clinics had attempted to comply with ethical codes, but they still tended to take training courses to learn further ethical codes [13]. To improve midwifery professional codes of ethics compliance and applicability rate, it seems of utmost importance to pay further attention to training and counseling these codes [9]. Counseling as an inevitable requirement in today´s world is a process that can lead to changes in perceptions, beliefs, attitudes, behaviors, and ultimately lifestyles [14, 15]. In a counseling process, counselors can thus make use a range of counseling methods (individual and group) to help clients make practical changes in different aspects of their lives and careers [16]. In this regard, Namadi *et al*. (2018) demonstrated that nursing ethics education through a case-based method could be an effective strategy for improving moral sensitivity among nursing students. It could also have a significant role in developing effective and lasting learning, helping in decision-making and improving quality of nursing care [17]. Baykara *et al*. (2015) had similarly concluded that ethics training could have a positive effect on improving moral sensitivity as well as ability to recognize ethical violations among nursing students [18]. Therefore, based on the existing scientific literature on midwifery professional codes of ethics as well as their sensitivity and importance in promoting this field and in order to provide better quality services to clients, this study investigated the effects of counseling professional ethics principles on midwifery professional codes of ethics compliance and applicability rate among midwives working in community health centers in the city of Karaj, Iran.

## Methods

### Sample selection

**Figure 1:**
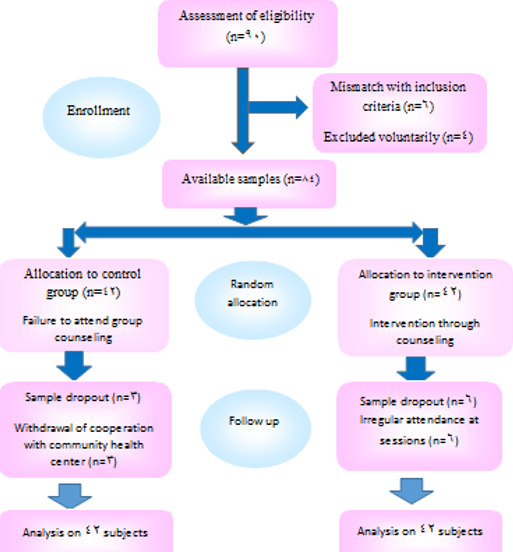
CONSORT flow diagram of patients in each stage of the RCT

This randomized controlled trial (RCT) was conducted on a population of 84 midwives working in community health centers in the city of Karaj in two intervention and control groups (42 individuals in each group), enrolled from July 23 to November 21, 2018; using multistage cluster sampling method. In the first phase of the sampling, the city of Karaj was first subdivided into northern, central, and southern regions. Then, a community health center was randomly selected from each region as the intervention group, and the closest center was considered as the control one. Random numbers were then sealed in a predetermined computer-made randomization opaque envelope. Accordingly, the participants were allocated to an intervention and a control group. The sampling was further continued to obtain the desired samples (14 in each selected region). The Consolidated Standards of Reporting Trials (CONSORT) flow diagram of the study is described in Figure 1. The samples were voluntarily enrolled on the basis of inclusion criteria i.e. a Bachelor´s degree or higher in midwifery, at least one year of work experience related to midwifery, failure to attend professional ethics-related workshops for at least six months before, and no history of referrals to the Islamic Republic of Iran Medical Council (IRIMC) and other relevant authorities to explain professional practices or to dismiss charges. The exclusion criterion was refusal to attend more than one in-person counseling session in the intervention group.

### Sample size

The sample size was estimated to be 33 in each group according to the study of Rahimparvar *et al*. (2014) that achieved a standard deviation of 0.552 for the professional codes of ethics in total [9], assuming counseling results in a half-unit increase and equality of variances between the intervention and control groups with 99% confidence interval and 90% test power using the following equation:

$$n=\frac{(s_{1}{}^{2}+s_{2}{}^{2}){\left(z_{1-\frac{a}{2}}+z_{1-\beta }\right)}^{2}}{{\left(\bar{x_{1}}-\bar{x_{2}}\right)}^{2}}$$

n = [(0.522^2^+0.522^2^)(2.58+1.28)^2^/0.5^2^] = 33. Considering the 20% dropout during the intervention, the final sample size was calculated by 42 in each group.

### Study protocol

The intervention group was initially provided with a training manual on professional midwifery ethics, then four 60-minute sessions of group counseling in a 14-person group and two 30-minute non-attendance sessions (Telegram social media) were held on a weekly basis in coordination with members and through notifications in advance on certain days and times. The design of counseling sessions in the intervention group is described in Table 1. A Telegram channel, controlled only by the researcher who had access to information, was created for the intervention group and it was loaded with midwifery professional codes of ethics counseling content. To ensure that the content of the Telegram counseling sessions had been received, the researcher posed questions about each session and presented them to each participant privately in cyberspace. New content was posted on the channel after making sure that the content of the previous session had been received. The counseling was also provided by the Telegram channel in audio, video, and text files. During the counseling, there were attempts to get people involved in discussions and comments, either in written or in audio forms, to the administrator privately in cyberspace, with questions being answered in the channel to address other member´ ambiguities. The researcher followed up and answered the questions from the intervention group members at the intervals of the counseling sessions and up to four weeks after completion of the sessions. Moreover, the participants were informed and reminded of the sessions to be held and the questionnaires to be completed by the Telegram channel and also via phone calls. It should be noted that in-person counseling sessions were conducted by a trained midwife practitioner in out-of-office hours in coordination with group members. Additionally, the training manual of the control group was distributed as the intervention group began counseling sessions to make them familiar with the 85 codes of ethics in midwifery profession in Iran. The Self-Reporting Questionnaire of Ethical Codes of Reproductive Health Providers was also completed in two groups before, immediately, and four weeks after the last counseling session. To comply with the principles of ethics at the end of the counseling sessions of the intervention group, a voluntary question-and-answer (Q&A) session was held for the control group.

**Table 1:** Content of counseling sessions for intervention group

Session no.	Attendance/ non-attendance in sessions	Session content
1	Attendance	Exploring study objectives, completing informed consent form and related questionnaires, providing a training manual on an overview of 85 midwifery professional codes of ethics in Iran and discussing generality of codes of ethics
2	Non-attendance	Uploading educational videos on importance of familiarity with codes of ethics in medical fields, uploading text files on 85 midwifery professional codes of ethics on the Telegram channel and requesting members by the channel administrator to see and study the content and ask any questions in case of ambiguities
3	Attendance	Education, counseling, group discussions, and role-plays in the first seven domains of the questionnaire of ethical codes of reproductive health providers used in this study
4	Attendance	Education, counseling, group discussions, and role-plays in the second seven domains of questionnaire of ethical codes of reproductive health providers used in this study
5	Non-attendance	Uploading scenarios related to 14 domains mentioned in the third and fourth sessions on the Telegram channel and asking members to leave their comments on the administrator page via announcing the number of each scenario
6	Attendance	Conducting a group discussion on scenarios presented in the fifth session, summarizing importance of compliance with midwifery professional codes of ethics and its impact on quality of services provided to clients, answering members´ questions, completing the second-stage questionnaire at the end of the session, appreciating participants and presenting gifts to them

### Measurements

The data collection instrument was a two-part questionnaire; the first part was a demographic characteristics form and the second part was a survey of midwifery professional codes of ethics compliance and applicability rate adapted from the Self-Reporting Questionnaire of Ethical Codes of Reproductive Health Providers in 20 domains, designed and evaluated by FarajKhoda *et al*. (2012) in the form of a PhD dissertation on reproductive health in an exploratory study, whose psychometric evaluation had shown content validity of 0.94, consensus of expert panel members by 94%, internal consistency of 86%, and stability of 95% [19]. In this study, 14 domains were evaluated in 95 items including compliance with client´s human dignity (6 items), compliance with client´s right to make decisions (11 items), obtaining client´s informed consent (4 items), disclosure to client (6 items), keeping client´s information confidential and secrecy (8 items), disclosing client´s information (6 items), compliance with client´s privacy (8 items), principle of profitability to client (6 items), no harm to client (5 items), compliance with principle of justice (5 items), service provider relationship with colleagues (5 items), service provider relationship with community (4 items), duty of service provider (14 items), and compliance with professional codes by service provider (7 items). Both qualitative and quantitative methods were also used to assess the content validity of the questionnaire. To do so qualitatively, 10 professionals in reproductive health and professional ethics across Iran were asked to submit their correctional views in writing after careful study of the tool. After collecting expert opinions, necessary revisions in the questionnaire were considered. As well, quantitative content validity showed that all the items had a minimum content validity ratio (CVR), based on the Lawshe table (0.62), and all the items scored above content validity index (CVI) of 0.79, thereby indicating appropriateness. The reliability of the research instrument was also assessed using intracluster correlation coefficient (ICC), which was 0.87, indicating an acceptable consistency of the questionnaire. A 5-point Likert-type scale was also employed to measure the compliance of each ethical item (i.e. very low=1, low=2, moderate=3, high=4, and very high=5 levels of compliance). The applicability rate of each item was additionally measured using the same questionnaire and scale.

### Statistical methods

The data were analyzed using the IBM SPSS Statistics (version 22) software via descriptive and inferential statistics. The Kolmogorov-Smirnov (K-S) test was also performed to examine the normality of the data. Parametric (repeated measures analysis of variance (ANOVA)) and non-parametric tests (generalized estimating equation (GEE)) were further used for data with normal and non-normal distribution; respectively. Chi-square test and/or independence t-test were also applied to investigate the correlation between two qualitative variables or grouped quantitative variables. Moreover, p<0.05 was considered as the significance level. Statistical analysis was carried out with an intention-to-treat (ITT) approach as emphasized by the CONSORT statement. To improve the reporting of the quality of trials, this statement declares that the number of participants in each group must be analyzed based on ITT principles [20].

### Ethical considerations

The Ethics Committee of Alborz University of Medical Sciences approved this study with the ethics code no. IR.ABZUMS.REC.1397.010. The RCT registration no. was also IRCT20150209021020N2.

## Results

In terms of demographic characteristics, the largest age group in both study groups was under 30 years in the intervention (58.3%; n = 21) and control (48.7%; n = 19) groups. Most of the participants were also married in the intervention (61.1%; n = 22) and control (64.1%; n = 25) groups. As well, majority of them had a Bachelor´s degree in the intervention (94.4%; n = 34) and control (94.9%; n = 37) groups. Considering interest in midwifery, most of the participants responded Yes in the intervention (100%; n=36) and control (97.4%; n = 38) groups. With regard to job satisfaction, majority of the respondents stated No in the intervention (69.4%; n = 25) and control (74.4%; n = 29) groups. Most of these individuals were contract employees in the intervention (86.1%; n = 31) and control (92.3%; n = 36) groups. In terms of economic status, the highest income level was in the category of 3 million tomans in the intervention group (27.8%; n=10) and 2 million tomans in the control group (23.1%; n = 9). The most frequent work experience in midwifery was under 3 years in the intervention group (36.1%; n = 13) and between 4 and 6 years in the control group (38.5%; n = 15). In terms of time elapsed from graduation, the longest time was 4-6 years in the intervention group (33.3%; n=12), and jointly two time periods below 3 years (30.8%; n = 12) and 4-6 years (30.8%; n = 12) were reported as the most frequent ones in the control group. According to the demographic data, the midwives working in community health centers in the city of Karaj showed no statistically significant differences in terms of age, marital status, level of education, interest in midwifery, job satisfaction, employment status, economic status, work experience in midwifery, as well as time elapsed from graduation. The participants in the intervention and control groups were thus homogenous regarding such demographic variables. In view of that, the results obtained after the intervention could be more confidently attributed to the effect of the intervention.

Based on the results of the Kolmogorov-Smirnov test examining normality of research variables, only two of the 14 domains of professional codes of ethics had non-normal distribution (i.e. service provider relationship with colleagues and service provider relationship with community). Therefore, these domains were analyzed via non-parametric tests, but other variables benefitted from normal distribution. According to the findings, compliance and applicability rate in all 14 domains of professional codes of ethics did not differ significantly in both groups before the intervention (p>0.05); however, there was a statistically significant post-intervention difference between the study groups (p < 0.05) (Table 2, Table 3). Furthermore, the results demonstrated that compliance and applicability rate in all 14 domains of midwifery professional codes of ethics were higher in the intervention group compared with the control one (Table 4, Table 5). As well, the findings of the statistical tests revealed that the trend of time changes in the mean compliance and applicability rate for the codes of all ethical domains during the three time points were significantly different between the two groups, and the highest level of compliance and applicability rate with the codes in both groups was related to the second time point (that is, immediately after the intervention).

**Table 2:** Comparison of level of compliance with professional codes of ethics in each domain between both study groups

Domains	Groups	Before intervention Mean±SD	Immediately after intervention Mean±SD	Paired t-test	Four weeks after intervention Mean±SD	Paired t-test
Compliance with client´s human dignity	Control	16.28±2.60	16.58±2.77	p=0.001 t=-3.69	16.12±2.5	p=0.02 t=1.29
	Intervention	17.11±2.74	18.86±1.22	p=0.001 t=-4.93	18.36±1.2	p=0.002 t=-3.44
Compliance with client´s right to make decisions	Control	37.46±4.71	38.02±4.97	p=0.001 t=-4.13	37.38±4.76	p=0.63 t=0.48
	Intervention	36.88±5.68	41.61±2.82	p=0.001 t=-7.15	40.94±2.74	p=0.001 t=-6.12
Obtaining client´s informed consent	Control	8.15±1.54	8.69±1.45	p=0.001 t=-4.45	8.30±1.30	p=0.32 t=-1.00
	Intervention	8.47±1.68	9.50±0.77	p=0.001 t=-4.46	9.25±0.76	p=0.003 t=-3.25
Disclosure to client	Control	15.84±2.35	16.38±2.44	p=0.001 t=-5.6	15.74±2.32	p=0.5 t=0.68
	Intervention	16.80±2.33	18.77±1.37	p=0.001 t=-6.21	18.30±1.30	p=0.001 t=-4.84
Keeping client´s information confidential and secrecy	Control	24.53±3.76	25.05±4.02	p=0.001 t=-4.44	24.12±3.68	p=0.006 t=2.91
	Intervention	25.16±4.10	28.00±2.16	p=0.001 t=-6.04	27.38±2.16	p=0.001 t=-4.68
Disclosing client´s information	Control	16.05±3.06	16.15±3.13	p=0.10 t=-1.67	15.79±3.03	p=0.003 t=3.21
	Intervention	16.36±3.29	18.63±1.45	p=0.001 t=-4.89	18.19±1.47	p=0.001 t=-3.92
Compliance with client´s privacy	Control	24.10±3.82	24.53±4.05	p=0.001 t=-3.79	23.92±3.83	p=0.164 t=1.42
	Intervention	25.52±4.26	28.13±2.09	p=0.001 t=-4.95	27.44±2.04	p=0.002 t=-3.32
Principle of profitability to client	Control	16.43±2.74	16.58±2.79	p=0.03 t=-2.23	15.87±2.80	p=0.001 t=5.5
	Intervention	17.33±2.57	18.80±1.43	p=0.001 t=-4.87	18.38±1.29	p=0.003 t=-3.17
No harm to client	Control	11.76±2.05	11.97±2.13	p=0.003 t=-3.13	11.41±2.03	p=0.001 t=4.17
	Intervention	11.63±2.75	13.83±1.40	p=0.001 t=-6.69	13.50±1.34	p=0.001 t=-5.43
Compliance with principle of justice	Control	12.28±1.93	12.64±1.96	p=0.001 t=-4.17	12.25±1.98	p=0.83 t=0.21
	Intervention	12.60±2.62	14.05±1.37	p=0.001 t=-3.53	13.65±1.32	p=0.01 t=-2.57
Service provider relationship with colleagues	Control	12.84±2.10	12.94±2.15	p=0.31 t=0.75	12.51±2.17	p=0.44 t=-0.76
	Intervention	13.02±2.44	14.36±0.93	p=0.02 t=-0.02	13.94±0.95	p=0.54 t=-0.61
Service provider relationship with community	Control	8.20±1.59	8.38±1.67	p=0.52 t=-0.63	8.00±1.65	p=0.58 t=-0.55
	Intervention	8.63±1.55	9.50±0.81	p=0.01 t=-2.37	9.00±0.92	p=0.67 t=-0.43
Duty of service provider	Control	44.48±6.32	45.71±7.14	p=0.001 t=-5.33	43.89±6.91	p=0.016 t=2.52
	Intervention	46.08±7.09	52.00±3.32	p=0.001 t=-6.75	51.05±3.04	p=0.001 t=-5.44
Compliance with professional codes by service provider	Control	20.97±3.41	21.41±3.56	p=0.001 t=-4.25	20.89±3.41	p=0.58 t=0.55
	Intervention	20.86±3.51	23.69±1.68	p=0.001 t=-6.37	22.94±1.63	p=0.001 t=-4.22

**Table 3:** Comparison of applicability rate of professional codes of ethics in each domain between both study groups

Domains	Groups	Before intervention Mean±SD	Immediately after the intervention Mean±SD	Paired t-test	Four weeks after intervention Mean±SD	Paired t-test
**Compliance with client´s human dignity**	Control	16.33±2.36	16.64±2.60	p=0.002 t=-3.38	15.51±2.42	p=0.001 t=3.45
	Intervention	17.00±2.76	18.72±1.78	p=0.001 t=-3.87	18.16±1.71	p=0.01 t=-2.66
**Compliance with client´s right to make decisions**	Control	36.33±4.98	36.89±5.22	p=0.001 t=-5.89	35.58±4.74	p=0.005 t=2.99
	Intervention	36.16±6.46	41.55±3.52	p=0.001 t=-5.39	40.91±3.41	p=0.001 t=-4.78
**Obtaining client´s informed consent**	Control	7.30±1.55	7.66±1.54	p=0.001 t=-3.84	7.20±1.64	p=0.55 t=0.6
	Intervention	8.33±1.70	9.25±1.10	p=0.001 t=-3.98	8.91±1.05	p=0.02 t=-2.33
**Disclosure to client**	Control	15.69±2.52	15.89±2.51	p=0.003 t=-3.13	15.12±2.28	p=0.001 t=4.13
	Intervention	16.00±3.05	18.33±1.75	p=0.001 t=-5.16	17.97±1.69	p=0.001 t=-4.32
**Keeping client´s information confidential and secrecy**	Control	24.23±3.52	24.79±3.71	p=0.001 t=-3.99	23.87±3.39	p=0.08 t=1.8
	Intervention	24.91±4.71	27.69±2.86	p=0.001 t=-4.09	27.16±2.81	p=0.002 t=-3.35
**Disclosing client´s information**	Control	16.17±2.59	16.41±2.69	p=0.002 t=-3.38	15.87±2.68	p=0.02 t=2.31
	Intervention	16.55±3.31	18.63±1.80	p=0.001 t=-4.26	18.38±1.80	p=0.001 t=-3.79
**Compliance with client´s privacy**	Control	24.84±3.46	25.33±3.68	p=0.001 t=-4.22	24.35±3.61	p=0.006 t=2.9
	Intervention	24.63±5.11	27.86±2.77	p=0.001 t=-4.31	27.66±2.68	p=0.001 t=-4.0
**Principle of profitability to client**	Control	16.58±2.56	16.89±2.55	p=0.001 t=-4.11	16.30±2.30	p=0.03 t=2.22
	Intervention	17.86±8.78	18.52±1.90	p=0.05 t=-0.43	18.08±1.79	p=0.88 t=-0.15
**No harm to client**	Control	11.71±1.94	12.15±2.17	p=0.001 t=-4.25	11.53±1.97	p=0.10 t=1.64
	Intervention	11.66±2.70	13.44±1.46	p=0.001 t=-4.7	12.94±1.67	p=0.004 t=-3.08
**Compliance with principle of justice**	Control	12.28±1.93	12.64±1.96	p=0.001 t=4.17	12.25±1.98	p=0.83 t=0.20
	Intervention	12.60±2.62	14.05±1.37	p=0.001 t=-3.53	13.65±1.32	p=0.01 t=-2.57
**Service provider relationship with colleagues**	Control	12.51±1.80	12.76±1.88	p=0.46 t=-0.73	12.33±1.76	p=0.53 t=-0.63
	Intervention	12.86±2.65	14.08±1.36	p=0.05 t=-0.34	13.83±1.25	p=0.53 t=-0.62
**Service provider relationship with community**	Control	8.15±1.36	8.53±1.57	p=0.17 t=-1.35	7.94±1.53	p=0.56 t=-0.57
	Intervention	8.52±1.57	9.36±1.09	p=0.016 t=-2.41	9.11±1.03	p=0.2 t=-1.27
**Duty of service provider**	Control	45.05±6.08	45.74±6.60	p=0.001 t=-4.84	44.74±6.47	p=0.07 t=1.82
	Intervention	46.13±7.48	51.27±4.72	p=0.001 t=-4.45	50.02±4.44	p=0.002 t=-3.39
**Compliance with professional codes by service provider**	Control	21.28±2.66	21.64±2.85	p=0.001 t=-3.84	21.23±2.80	p=0.71 t=0.37
	intervention	20.51±4.25	23.45±2.11	p=0.001 t=-4.52	22.62±2.17	p=0.003 t=-3.17

**Table 4:** Comparison of level of compliance with professional codes of ethics in each domain in intervention and control groups at three time points

Domains	Groups	Before intervention Mean±SD	Immediately after intervention Mean±SD	Four weeks after intervention Mean±SD	Repeated measures ANOVA	
					Inter-group	Intra-group
**Compliance with client´s human dignity**	Control	16.28±2.60	16.58±2.77	16.12±2.5	F=4620.59 p<0.001	F=14.33 p<0.001
	Intervention	17.11±2.74	18.86±1.22	18.36±1.2		
**Compliance with client´s right to make decisions**	Control	37.46±4.71	38.02±4.97	37.38±4.76	F=6278.43 p<0.001	F=39.37 p<0.001
	Intervention	36.88±5.68	41.61±2.82	40.94±2.74		
**Obtaining client´s informed consent**	Control	8.15±1.54	8.69±1.45	8.30±1.30	F=4086.80 p<0.001	F=4.95 P=0.02
	Intervention	8.47±1.68	9.50±0.77	9.25±0.76		
**Disclosure to client**	Control	15.84±2.35	16.38±2.44	15.74±2.32	F=5542.08 p<0.001	F=22.70 p<0.001
	Intervention	16.80±2.33	18.77±1.37	18.30±1.30		
**Keeping client´s information confidential and secrecy**	Control	24.53±3.76	25.05±4.02	24.12±3.68	F=4572.68 p<0.001	F=30.19 p<0.001
	Intervention	25.16±4.10	28.00±2.16	27.38±2.16		
**Disclosing client´s information**	Control	16.05±3.06	16.15±3.13	15.79±3.03	F=3294.53 p<0.001	F=20.98 p<0.001
	Intervention	16.36±3.29	18.63±1.45	18.19±1.47		
**Compliance with client´s privacy**	Control	24.10±3.82	24.53±4.05	23.92±3.83	F=4494.09 p<0.001	F=13.50 p<0.001
	Intervention	25.52±4.26	28.13±2.09	27.44±2.04		
**Principle of profitability to client**	Control	16.43±2.74	16.58±2.79	15.87±2.80	F=4253.60 p<0.001	F=23.05 p<0.001
	Intervention	17.33±2.57	18.80±1.43	18.38±1.29		
**No harm to client**	Control	11.76±2.05	11.97±2.13	11.41±2.03	F=3195.16 p<0.001	F=42.28 p<0.001
	Intervention	11.63±2.75	13.83±1.40	13.50±1.34		
**Compliance with principle of justice**	Control	12.28±1.93	12.64±1.96	12.25±1.98	F=4129.71 p<0.001	F=6.92 P=.010
	Intervention	12.60±2.62	14.05±1.37	13.65±1.32		
**Service provider relationship with colleagues**	Control	12.84±2.10	12.94±2.15	12.51±2.17	Wald Chi-Square=12.51 Df=1 P=0.001	
	Intervention	13.02±2.44	14.36±0.93	13.94±0.95		
**Service provider relationship with community**	Control	8.20±1.59	8.38±1.67	8.00±1.65	Wald Chi-Square=5.19 Df=1 P=0.023	
	Intervention	8.63±1.55	9.50±0.81	9.00±0.92		
**Duty of service provider**	Control	44.48±6.32	45.71±7.14	43.89±6.91	F=5217.67 p<0.001	F=37.26 p<0.001
	Intervention	46.08±7.09	52.00±3.32	51.05±3.04		
**Compliance with professional codes by service provider**	Control	20.97±3.41	21.41±3.56	20.89±3.41	F=4363.26 p<0.001	F=19.00 p<0.001
Intervention	20.86±3.51	23.69±1.68	22.94±1.63			

**Table 5:** Comparison of applicability rate of professional codes of ethics in each domain in intervention and control groups at three time points

Domains	Groups	Before intervention Mean±SD	Immediately after intervention Mean±SD	Four weeks after intervention Mean±SD	Repeated measures ANOVA	
					Inter- group	Intra-group
**Compliance with client´s human dignity**	Control	16.33±2.36	16.64±2.60	15.51±2.42	F=5009.17 p<0.001	F=16.51 p<0.001
	Intervention	17.00±2.76	18.72±1.78	18.16±1.71		
**Compliance with client´s right to make decisions**	Control	36.33±4.98	36.89±5.22	35.58±4.74	F=5559.24 p<0.001	F=30.91 p<0.001
	Intervention	36.16±6.46	41.55±3.52	40.91±3.41		
**Obtaining client´s informed consent**	Control	7.30±1.55	7.66±1.54	7.20±1.64	F=2770.77 p<0.001	F=5.25 P=0.02
	Intervention	8.33±1.70	9.25±1.10	8.91±1.05		
**Disclosure to client**	Control	15.69±2.52	15.89±2.51	15.12±2.28	F=4376.89 p<0.001	F=30.25 p<0.001
	Intervention	16.00±3.05	18.33±1.75	17.97±1.69		
**Keeping client´s information confidential and secrecy**	Control	24.23±3.52	24.79±3.71	23.87±3.39	F=4531.29 p<0.001	F=14.81 p<0.001
	Intervention	24.91±4.71	27.69±2.86	27.16±2.81		
**Disclosing client´s information**	Control	16.17±2.59	16.41±2.69	15.87±2.68	F=3952.34 p<0.001	F=19.49 p<0.001
	Intervention	16.55±3.31	18.63±1.80	18.38±1.80		
**Compliance with client´s privacy**	Control	24.84±3.46	25.33±3.68	24.35±3.61	F=4538.39 p<0.001	F=22.13 p<0.001
	Intervention	24.63±5.11	27.86±2.77	27.66±2.68		
**Principle of profitability to client**	Control	16.58±2.56	16.89±2.55	16.30±2.30	F=3036.11 p<0.001	F=0.11 P=0.734
	Intervention	17.86±8.78	18.52±1.90	18.08±1.79		
**No harm to client**	Control	11.71±1.94	12.15±2.17	11.53±1.97	F=3296.630 p<0.001	F=12.351 P=0.001
	Intervention	11.66±2.70	13.44±1.46	12.94±1.67		
**Compliance with principle of justice**	Control	12.28±1.93	12.64±1.96	12.25±1.98	F=4129.71 p<0.001	F=6.92 P=0.010
	Intervention	12.60±2.62	14.05±1.37	13.65±1.32		
**Service provider relationship with colleagues**	Control	12.51±1.80	12.76±1.88	12.33±1.76	Wald Chi-Square=8.85 Df=1 P=0.003	
	Intervention	12.86±2.65	14.08±1.36	13.83±1.25		
**Service provider relationship with community**	Control	8.15±1.36	8.53±1.57	7.94±1.53	Wald Chi-Square=7.51 Df=1 P=0.006	
	Intervention	8.52±1.57	9.36±1.09	9.11±1.03		
**Duty of service provider**	Control	45.05±6.08	45.74±6.60	44.74±6.47	F=5262.07 p<0.001	F=14.12 p<0.001
	Intervention	46.13±7.48	51.27±4.72	50.02±4.44		
**Compliance with professional codes by service provider**	Control	21.28±2.66	21.64±2.85	21.23±2.80	F=5273.76 p<0.001	F=11.17 P=0.001
	Intervention	20.51±4.25	23.45±2.11	22.62±2.17		

## Discussion

The results of the present study established that counseling professional ethics principles had an effect on level of compliance and applicability rate of midwifery professional codes of ethics (in all 14 domains investigated) among the midwives working in community health centers. It should be further noted that searches in different databases revealed that most previous studies had a descriptive design, and there was a lack of interventional research in this area. Additionally, the study settings in most of investigations, somehow related to the main concepts of the present study, were hospitals, which was itself a factor in making a difference in the reported findings. Accordingly, Rahimparvar *et al*. (2014) had examined level of compliance with professional codes of ethics in midwives working in the city of Tehran and had found that the lowest mean score in this regard was related to the domain of disclosure of client´s information and the highest one was associated with the domain of professional relationship with colleagues [9]. The results of the study by Shaali *et al*. (2018) had also indicated no significant difference in compliance with professional codes of ethics (self-reporting) in midwives working in gynecology wards (85.1±12.02) and those involved in maternity wards affiliated to Isfahan University of Medical Sciences (80.5±14.7) [21]. Besides, Yousefzadeh *et al*. (2015) had determined that the mean practice of midwives working in maternity wards of teaching hospitals in the city of Mashhad with regard to professional codes of ethics was 61.8±9.7 (based on a score of 0 to 100) using a checklist observed by the researcher [22]. Masumi *et al*. (2016) had similarly reported that the highest level of compliance with professional codes of ethics among midwifery students was respect for client´s rights, professional commitments, communication with other students, communication with clients and caregivers, ethics in research and education, and ethics in management [10]. Evaluation of the results of the above descriptive studies mainly suggested moderate level of compliance with professional codes of ethics in midwifery staff or students, indicating needs for professional ethics retraining courses to improve quality of reproductive health services in communities. It also showed the necessity of conducting the present study. Moreover, methods of evaluation (self-reporting) for level of compliance with professional codes of ethics in most studies were similar to that utilized in the present study.

Besides, the results of an investigation by Azizi *et al*. (2016) on the effect of nursing ethics education on moral judgment of nurses had revealed a significant decrease in mean scores of moral judgment in the intervention group after four 90-minute sessions, so that the nursing ethics education had significantly enhanced moral judgment of nurses in the intervention group [23]. Yarbrough *et al*. (2007) had also argued that discussing ethical dilemmas associated with clinical practices had helped nurses better identify ethical problems and make appropriately ethical decisions by thinking about them and applying ethical principles [24]. Moreover, Grady *et al*. (2008) had stated that ethics education could play an important role in augmenting nurses´ moral judgment [25]. The results of a study by Khaje Mozaffari *et al*. (2017), entitled “Effect of educational intervention on compliance with medical ethics in morning report sessions”, had shown that the education program (i.e. a 12-hour workshop and morning report sessions by ethics professors for 3 months) had led the students to introduce patients almost with the preservation of human dignity, and ultimately to increase the accuracy of their introduction. After training, the items including no emotional harm, no violation of privacy, compliance with general principles of data collection and history taking, and introduction of patients without expressing identity information had been observed in more than 75% of cases. Therefore, training in medical ethics had significantly enhanced students´ attitudes towards quality of morning reporting sessions [26]. The study by Hassanpoor *et al*. (2011) in the form of teaching nursing ethics (four 4-hour sessions per week) on moral sensitivity had correspondingly revealed a significant difference in the mean moral sensitivity in nurses´ decision-making between pre- and post-intervention groups, indicating the effect of nursing ethics education on moral sensitivity in nurses´ decision-making [27]. Hosseini *et al*. (2018) in another study on the effect of moral motivation training (i.e. a workshop for two days and 5 hours each day) on moral sensitivity in nurses of military hospitals had correspondingly found that the moral motivation training as a combination of lectures, group discussions, movie screening, and role-plays had significantly contributed to improving nurses´ moral sensitivity [28]. The results of a study by Imanifar *et al*. (2015), entitled “Comparing the effect of teaching ethical principles using narrative ethics and lectures (four 3-hour sessions per week for both groups) on moral sensitivity of nurses”, had further demonstrated that both narrative ethics and lectures had significantly increased nurses´ moral sensitivity in both groups before and after the intervention, despite the higher mean scores of changes in the moral sensitivity of the narrative ethics group. As well, comparison between the two groups had shown no significant difference. The mean score of moral sensitivity has also decreased significantly three months after the intervention in both groups [29]. The findings from a study by Ebrahimi *et al*. (2017) on the impact of ethics workshop on ethical knowledge and competence of fourth-year medical students of Shiraz University of Medical Sciences had similarly revealed a statistically significant difference in importance of ethical components between pre- and post-attendance in medical ethics workshops, emphasizing the role of education in understanding the importance of medical ethics components, especially in subjects such as secrecy and informed consent for students [30].

The results of all these studies established that educational interventions of professional codes of ethics had a significant effect on improvement of ethical compliance, moral judgment, and moral sensitivity, which were consistent with the findings of the present study. Since the study consisted of four attendance sessions and two non-attendance sessions (6 sessions in total), and a set of methods such as education, counseling, group discussions, role- plays, as well as audio, video and text files exchanged through presence of all members in a Telegram channel, and there had been attempts to argue with participants, who had channeled their ideas in written or audio forms in cyberspace specifically for the administrator during counseling, the effects of counseling professional ethics principles seemed to be significant, particularly in the immediate post-intervention period. Failure to follow up the participants over the long term could also justify the lower mean score of compliance and applicability rate of codes four weeks after the intervention compared with immediately after it. Some of the limitations of the present study were the self-reporting data collection method and short post-intervention period (four weeks) for conclusive statements. Leakage of session contents from the intervention group into the control group could be also among the limitations of this study, resolved by changing the community health centers for the control group.

## Conclusion

Compliance with principles of professional ethics can help protect midwifery and midwives from the potentially unpleasant consequences of some professional challenges, improve quality of services provided, promote client´s level of satisfaction, and increase public confidence in this profession. Therefore, the greater value should be given to training professional ethics in schools of midwifery and related workplaces and consequently midwives´ performance should be evaluated in this area to identify weaknesses and to take positive actions to address such shortcomings [31, 32].

### What is known about this topic

- In recent years, attention to ethical principles in various medical professions including midwifery has become of utmost importance;
- Most of descriptive studies fulfilled in this respect have suggested that the required policies (such as educational interventions) should be designed in order to enhance more compliance with codes of ethics and consequently improve quality of clinical services.

### What this study adds

- Holding professional ethics counseling sessions such as educational sessions can promote compliance and applicability rate of midwifery professional codes of ethics;
- The highest level of compliance and applicability rate of the codes in the intervention and control groups were related to those immediately after the intervention, not four weeks after it;
- Over time, the effects of professional ethics counseling sessions had diminished.

## Competing interests

The authors declare no competing interests.

## References

[1] Khodadost K, Hosini ST, Mohjal shoja MA (2010). Medical ethics and its importance in ancient Iran and Islam. ijme.

[2] Mahmoodi Shan G, Alhani F, Ahmadi F, Kazemnejad A (2009). Ethics in nurses' lifestyle: a qualitative study. ijme.

[3] Fazeli Z, Fazeli Bavandpour F, Rezaee Tavirani M, Mozafari M, Haidari Moghadam R (2013). Professional ethics and its role in the medicin. sjimu.

[4] Horton K, Tschudin V, Forget A (2007). The value of nursing: a literature review. Nursing ethics.

[5] Maaarefi F, Eslamiakbar R, Ashktorab T, Abaszade A, Alavimajd H (2014). Check the patient's perspective in relation to compliance with ethical codes of nursesé professional commitment in Medical Sciences hospitals of Jahrom in 2013. ECNM.

[6] Markose A, Krishnan R, Ramesh M (2016). Medical ethics. J Pharm Bioallied Sci.

[7] Azimi N, Kiani M, Amadi M, Alavimajd H (2014). Awareness of midwifery'students, medical interns women and gynecologist students about medical ethics standards in department of labor, chosen hospitals training of Tehran in 2011. Medical Ethics Journal.

[8] Attarha M, keshavarz z, Bakhtiari M, Jamilian M (2016). Explaination of the concept of midwife-mother relationship in delivery rooms: A qualitative content analysis. J Qual Res Health Sci.

[9] Vasegh Rahimparvar SF, Nasiriani L, Farajkhoda T, Bahrani N (2014). Compliance rate of midwives with the professional codes of ethics in Maternal Child Health Centers in Tehran. ijme.

[10] Masumi SZ, Golalizadeh Bibalan F, Roshanaei Gh (2016). Observance of midwifery code of ethics among midwifery students and its related factors. Medical Ethics Journal.

[11] Shahhoseini Z, Rashidi S, Abedian K (2005). Midwifery awareness of legal rules and drug use in midwifery. Ir J Forensic Med.

[12] Ghobadifar MA, Mosalanejad L (2013). Evaluation of staff adherence to professionalism in Jahrom University of Medical Sciences. J Educ Ethics Nurs.

[13] Turkmen AS, Savaser S (2015). Pediatric Nursesé Information and applications related to ethical codes. Iran J Pediatr.

[14] Parand A, Khodayarifard M (2012). Stress and coping methods with stress.

[15] PorAsghar M (2011). Counseling principles and guidelines in Islam. Marefat.

[16] Rezvani E The effect of health behavior counseling on self-care of women with pregnancy decision referring to the Jahanshahr Women's Park in Karaj.

[17] Namadi F, Hemmati Maslakpak M, Moradi Y, Ghasemzadeh N (2018). The effect of professional ethics education throgh case-based method on moral sensitivity in nursing students: a clinical trial study. J Urmia Nurs Midwifery Fac.

[18] Baykara ZG, Demir SG, Yaman S (2015). The effect of ethics training on students recognizing ethical violations and developing moral sensitivity. Nurs Ethics.

[19] FarajKhoda T (2012). Developing the professional codes of ethics for reproductive health care provider and ités assessment from their viewpoints in Yazd health care centers.

[20] Schulz KF, Altman DG, Moher D, CONSORT Group (2011). CONSORT 2010 statement: updated guidelines for reporting parallel group randomised trials. Int J Surg.

[21] Shaali M, Shahriari M, Abdishahshahan MI (2018). Evaluation of compliance rate of midwives with the codes of professional ethics in maternity and gynecology wards of hospitals affiliated with Isfahan University of Medical Sciences in 2016. IJOGI.

[22] Yousefzadeh S, Kordi M, Mazloum SR, Tara F (2015). The survey of midwivesé knowledge, attitude and practice about professional ethics codes in the maternity of Mashhad educational hospitals in 2014. IJOGI.

[23] Azizi A, Sepahvani M, Mohamadi J (2016). The effect of nursing ethics education on the moral judgment of nurses. JNE.

[24] Yarbrough S, Klotz L (2007). Incorporating cultural issues in education for ethical practice. Nursing ethics.

[25] Grady C, Danis M, Soeken KL, O'Donnell P, Taylor C, Farrar A (2008). Does ethics education influence the moral action of practicing nurses and social workers?. Am J Bioeth.

[26] Khaje Mozaffari J, Sohrab MB, Zaroug Hossaini R, Zolfaghari P, Najafi F, Yahyaei E (2017). The effect of educational intervention on the degree of compliance with standards of medical ethics in morning report sessions. Medical Ethics Journal.

[27] Hassanpoor M, Hosseini M, Fallahi Khoshknab M, Abbaszadeh A (2011). Evaluation of the impact of teaching nursing ethics on nurses' decision making in Kerman social welfare hospitals in 1389. ijme.

[28] Hoseini M, Ebadi M, Farsi Z (2018). The effect of moral motivation training on moral sensitivity in the nurses of Military Hospitals. Military Caring Sciences.

[29] Imanifar N, Vaghar Seyedin A, Afshar L, Sharifzadeh Gh (2015). Comparison effect of teaching ethical principles using narrative ethics and lecture on the morl sensitivity of nurses. Medical Ethics Journal.

[30] Ebrahimi S, Alinejad N (2017). The impact of ethics workshop on the ethical knowledge and competency of fourth years medical students of Shiraz University of Medical Sciences. ijme.

[31] Hasanpour azghadi S, Abbasi Z (2009). . Effect of education on middle-aged women's practice towards menopause. JNKUMS.

[32] Nasiriani L (2014). Compliance rate of professional codes of ethics in midwifery and its related factors among midwives of Maternal Child Health Centers in Tehran.

